# Low-Cost Active Thermography using Cellphone Infrared Cameras: from Early Detection of Dental Caries to Quantification of THC in Oral Fluid

**DOI:** 10.1038/s41598-020-64796-6

**Published:** 2020-05-12

**Authors:** Nakisa Samadi, Damber Thapa, Mohammadhossein Salimi, Artur Parkhimchyk, Nima Tabatabaei

**Affiliations:** Department of Mechanical Engineering, Lassonde School of Engineering, York University, 4700 Keele St., Toronto, ON M3J 1P3 Canada

**Keywords:** Biomedical engineering, Optical imaging, Optical sensors, Imaging techniques, Electrical and electronic engineering

## Abstract

Active thermography (AT) is a widely studied non-destructive testing method for the characterization and evaluation of biological and industrial materials. Despite its broad range of potential applications, commercialization and wide-spread adaption of AT has long been impeded by the cost and size of infrared (IR) cameras. In this paper, we demonstrate that this cost and size limitation can be overcome using cell-phone attachment IR cameras. A software development kit (SDK) is developed that controls camera attributes through a simple USB interface and acquires camera frames at a constant frame rate up to 33 fps. To demonstrate the performance of our low-cost AT system, we report and discuss our experimental results on two high impact potential applications. The first set of experiments is conducted on a dental sample to investigate the clinical potential of the developed low-cost technology for detecting early dental caries, while the second set of experiments is conducted on the oral-fluid based lateral flow immunoassay to determine the viability of our technology for detecting and quantifying cannabis consumption at the point-of-care. Our results suggest achievement of reliable performance in the low-cost platform, comparable to those of costly and bulky research-grade systems, paving the way for translation of AT techniques to market.

## Introduction

Over the past four decades, the notion of non-radiative conversion of light energy into heat energy (photothermal sciences) has made remarkable achievements in the development of metrology and imaging techniques in the fields of basic sciences, engineering, and medicine^1^. Many of such techniques utilize infrared (IR) thermography for detection of materials’ defects, or tissue malignancies, through interrogation of thermal radiations emitted from specimens in response to external excitations (aka. active thermography)^2^. The role of external excitation (normally optical excitation) in active thermography (AT) is to enable reliable detection of an *a priori* known waveform in the highly noisy thermal radiation signals of specimens. In the presence of defects, the thermal impedances introduced by defects alter the induced local temperature field which in return alters the amplitude and phase of the *a priori* known radiative signals captured from defective regions. Consequently, the demodulation of IR radiative signals enables the detection of defective regions with sharp contrast and high signal-to-noise ratio (SNR).

Based on the temporal pattern of external excitation, AT techniques can be categorized into pulsed thermography, lock-in thermography (LIT), and matched-filter thermography. In pulsed thermography, the sample is excited by a short pulse of light, usually a flash lamp, and the transient surface temperature profile is, subsequently, recorded by a thermal camera and then analyzed^3^. In LIT, on the other hand, a single-frequency intensity-modulated external excitation (e.g., laser) is utilized to generate a steady-state modulated temperature field (aka thermal-wave field) inside the sample while recording the sample temporal temperature responses with an IR camera^4–6^. A key shortcoming of both pulsed and LIT is the inherent compromise between inspection depth and depth resolution^2^. That is, in both methods, inspecting deep into the sample comes at the price of deterioration of the resolution of the imaging system due to the diffuse nature of thermal waves. To alleviate this shortcoming, recently, matched-filter thermography has been introduced as the photothermal analog of optical coherence tomography (OCT)^7–11^. In matched-filter thermography methods, radar pulsed-compression techniques, such as linear frequency modulation^7,8^ or binary phase coding^9^, are used for the external excitation in order to improve system point spread function to achieve depth-resolved and “crisp” images from an intrinsically diffuse thermal-wave field.

The non-contact and non-ionizing nature of AT, as well as its tunability to probe a broad range of materials (e.g., opaque^12–16^, turbid/biological^17–19^) using different types of excitation sources (e.g., optical^18^, magnetic^13^, mechanical waves^15^, electrical^12,20^ or even cyclic stress/strain^14^), have resulted in the widespread adoption of AT in non-destructive testing research and development. For example, AT techniques have been widely utilized by researchers for inspection of industrial samples for detection of damages in Carbon Fiber Reinforced Plastic (CFRP) materials^21^, inspection of airplane parts^22^ and detection of electric leakages in integrated circuits^20^. More recently, AT has been utilized to detect malignancies in human hard and soft tissues such as early detection of demineralization in dental enamel^17–19^, cutaneous melanoma^23^, mineral loss in bone^24^ or tumors^25^.

Despite the abovementioned broad range of applications, commercialization and wide-spread adoption of these techniques by industry have been significantly impeded by the cost and the size of IR cameras used. IR cameras suitable for thermal measurements are either mid-wavelength (MWIR: 3–5 µm) or long-wavelength (LWIR: 8–14 µm), measuring sample temperature based on objects’ thermal/Planck radiation^26^. MWIR cameras typically use sensitive photon detectors but need dedicated cooling systems which result in higher size, cost, weight, and complexity of the imaging system. LWIR cameras, on the other hand, use less sensitive uncooled microbolometer thermal detectors and can be produced at a lower cost compared to MWIR cameras^27,28^. In general, the price of research-grade thermal cameras, depending on the type (MWIR vs LWIR), number of pixels, objective focal length and F-number ranges between $10k–$200k, posing a key barrier to commercialization of AT technologies. In an attempt to overcome this barrier, we recently demonstrated the possibility of incorporating low-cost (~$250) and small cell-phone attachment LWIR camera instead of the costly research-grade IR cameras for performing AT^29^. In that initial work^29^, the performance of the developed LIT system was limited by the slow and inconsistent frame rate of the camera (~15 fps) as well as the disruptions in the image acquisition due to frequent execution of cell-phone attachment camera’s native calibration and non-uniformity correction procedures. Here in this manuscript, we report on the development of a reliable software development kit (SDK) which not only enables control of camera attributes but also offers reliable acquisition of frames at constant frame rates of up to 33 fps through a simple USB interface. To demonstrate the feasibility of conducting reliable AT with a low-cost cellphone IR camera, in sections below, after discussing the significance of performing LIT at high frame rates, we present and discuss our experimental results on two high impact areas of detecting early stages of demineralization in human dental enamel and detection and quantification of cannabis in oral fluid.

## Results and Discussions

### Evaluation of SDK and developed low-cost LIT system performance

**Figure 1:**
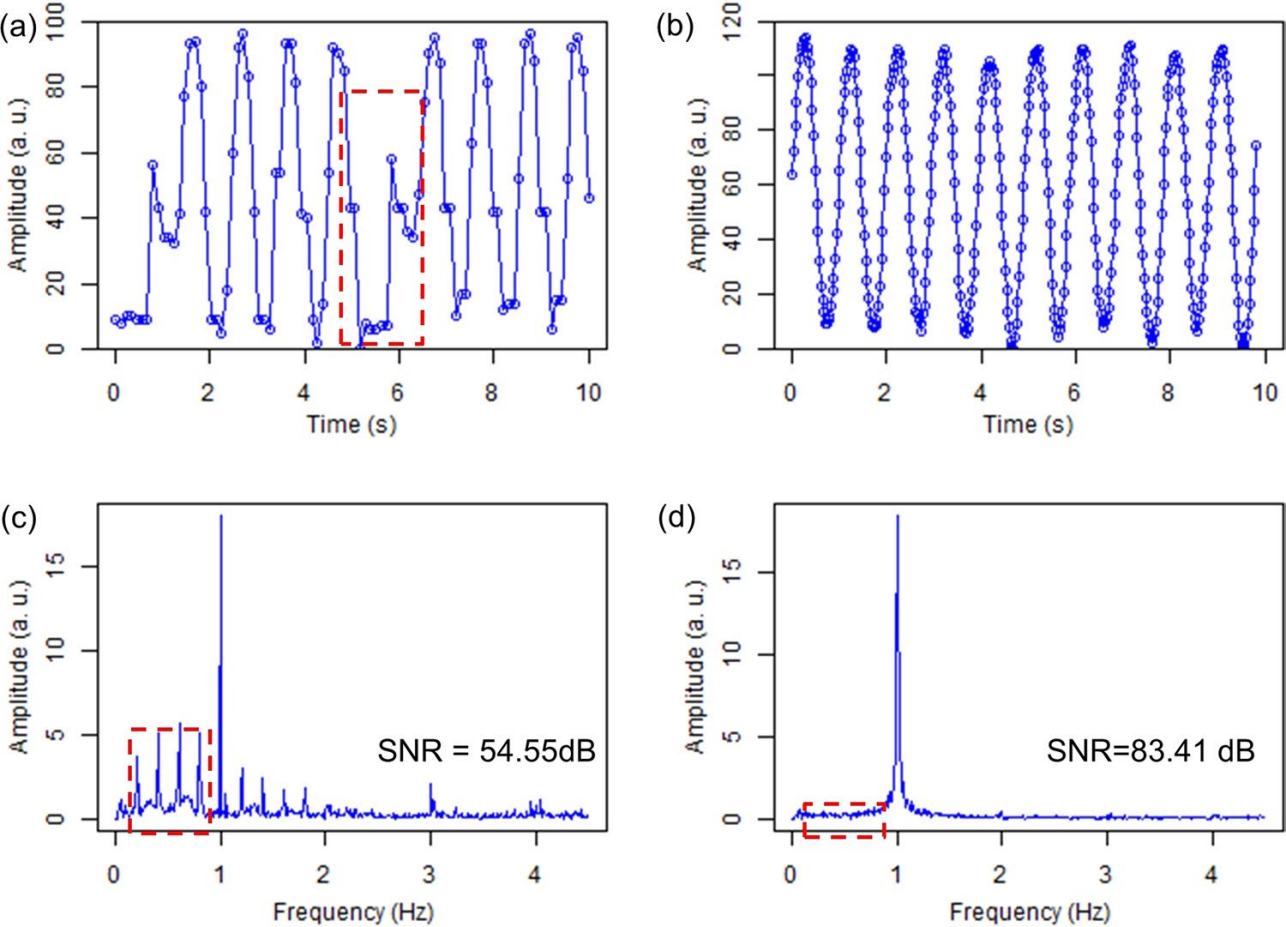
Temporal variations of surface temperature captured by (**a**) the SEEK THERMAL app and (**b**) developed SDK. (**c**,**d**) Are Fourier spectrums of (**a**,**b**), respectively.

To study the advantages and limitations of developed SDK platform over manufacturer’s software, comparative LIT experiments were carried out using the IR camera frames captured via developed SDK and those directly captured by the SEEK APP (Seek Thermal Inc.; Santa Barbara, CA) hosted on an Android Google Pixel 4 cellphone. LIT experiments were carried out on a thick Aluminum block (45 mm × 25 mm × 30 mm) interrogated at a laser modulation frequency of 1 Hz to compare the maximum frame rates and consistency of image acquisitions. Figure 1a,b show sections (10 sec) of the time-laps signals from the central pixel of the camera captured via manufacturer applet and developed SDK, respectively. These oscillations of surface temperature are the thermal waves recorded from the sample surface of a semi-infinite opaque body. Evaluation of these signals suggests periodic disruptions in the recording of the surface thermal wave by manufacturer’s software due to periodic execution of a non-uniformity correction routine, red dashed rectangle in Fig. 1(a). Signals captured by developed SDK, on the other hand, are continuous and without any disruption, resulting in the proper realization of the 1 Hz modulation frequency of the thermal-wave, Fig. 1(b). The maximum achievable frame rate is also significantly larger (33 Hz vs 9 Hz) with the SDK. Figure 1c,d show the spectra of the recorded waveforms in the Fourier domain. These spectra suggest that the presence of disruptions in the acquisition of frames by manufacturer software results in the appearance of erroneous frequency components in the frequency domain, while the spectrum of the signal acquired by developed SDK shows a single dominant peak at the modulation frequency of the laser. The inconsistent and slower frame acquisition with manufacturer software also results in lower SNR of recorded thermal-waves. The SNRs for the signals obtained under identical experimental conditions using manufacturer software and developed SDK were found as 54.55 dB and 83.41 dB, respectively (using equation (1); Methods section). These qualitative and quantitative comparisons of quality of signals suggest the superior and reliable performance of developed SDK, paving the way for the development of low-cost, yet reliable, AT systems and their commercialization. We intend to offer the developed SDK to the scientific and educational communities at no charge and in the context of collaboration; therefore, entities interested in using the SDK are urged to contact us.

**Figure 2:**
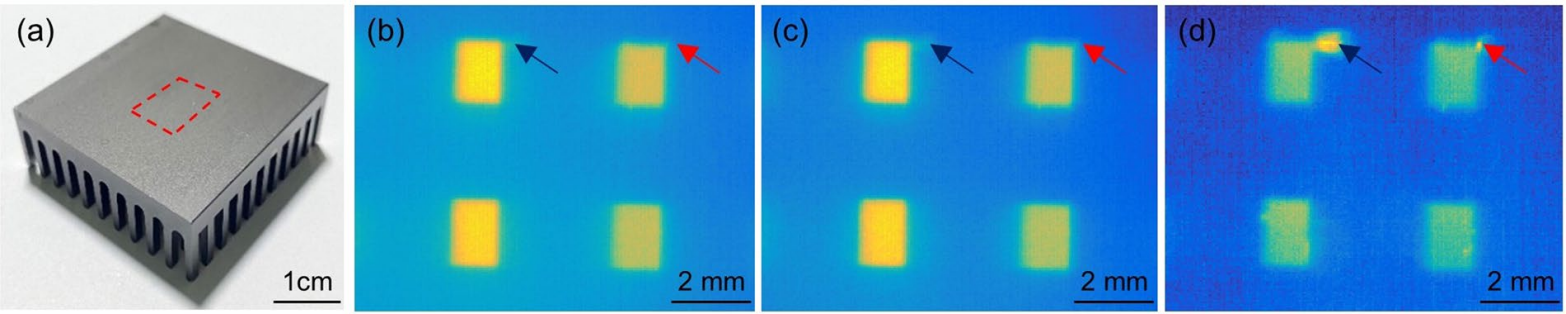
(**a**) Photograph of a thermal heat sink; red dashed rectangle depicts the imaging area. LIT Amplitude image of the area of heat sink imaged at modulation frequency of 1 Hz with camera frame rates of (**b**) 9 fps and (**c**) 33 fps. (**d**) LIT Amplitude image at a modulation frequency of 10 Hz and camera frame rate of 33 Hz, demonstrating the possibility of resolving small defects with the improved resolution at higher modulation frequencies with high frame rates.

As shown in Fig. 1, the developed SDK allows for enhancement of the acquisition rate from 9 fps to 33 fps. One of the benefits of having a higher frame rate in LIT is that it enables proper sampling of higher frequency thermal waves based on the Nyquist–Shannon sampling theorem. LIT at higher modulation frequencies results in shortening of thermal diffusion length. This shortening, in return, leads to improvement of image resolution, enabling the detection of smaller defects. To demonstrate this added value experimentally, we conducted LIT experiments on a pin-fin thermal heat sink, Fig. 2a. The size of the heat sink is 40.6 mm × 40.6 mm × 13.3 mm and consists of pin fins of cross-section size 1.4 mm × 2.4 mm at 2 mm beneath top plate. Figure 2b,c show LIT amplitude images at a modulation frequency of 1 Hz at frame rates of 9 fps and 33 fps, respectively. These images clearly show the location and size of the subsurface fins. However, no defect can be resolved at the connection sites between fins and plate based on images at 1 Hz modulation frequency, not even at 33 fps. Figure 2d is a LIT amplitude image at a laser modulation frequency of 10 Hz with a frame rate of 33fps; note that the native camera frame rate of 9 fps cannot sample a 10 Hz thermal wave according to Nyquist–Shannon sampling theorem. The advantage of higher frame rate and higher modulation frequency is clearly seen in the 10 Hz amplitude image. Small connection defects in the top two fins (arrows) can clearly be resolved due to the reduction of thermal diffusion length and corresponding improvement of resolution at the higher modulation frequency.

**Figure 3:**
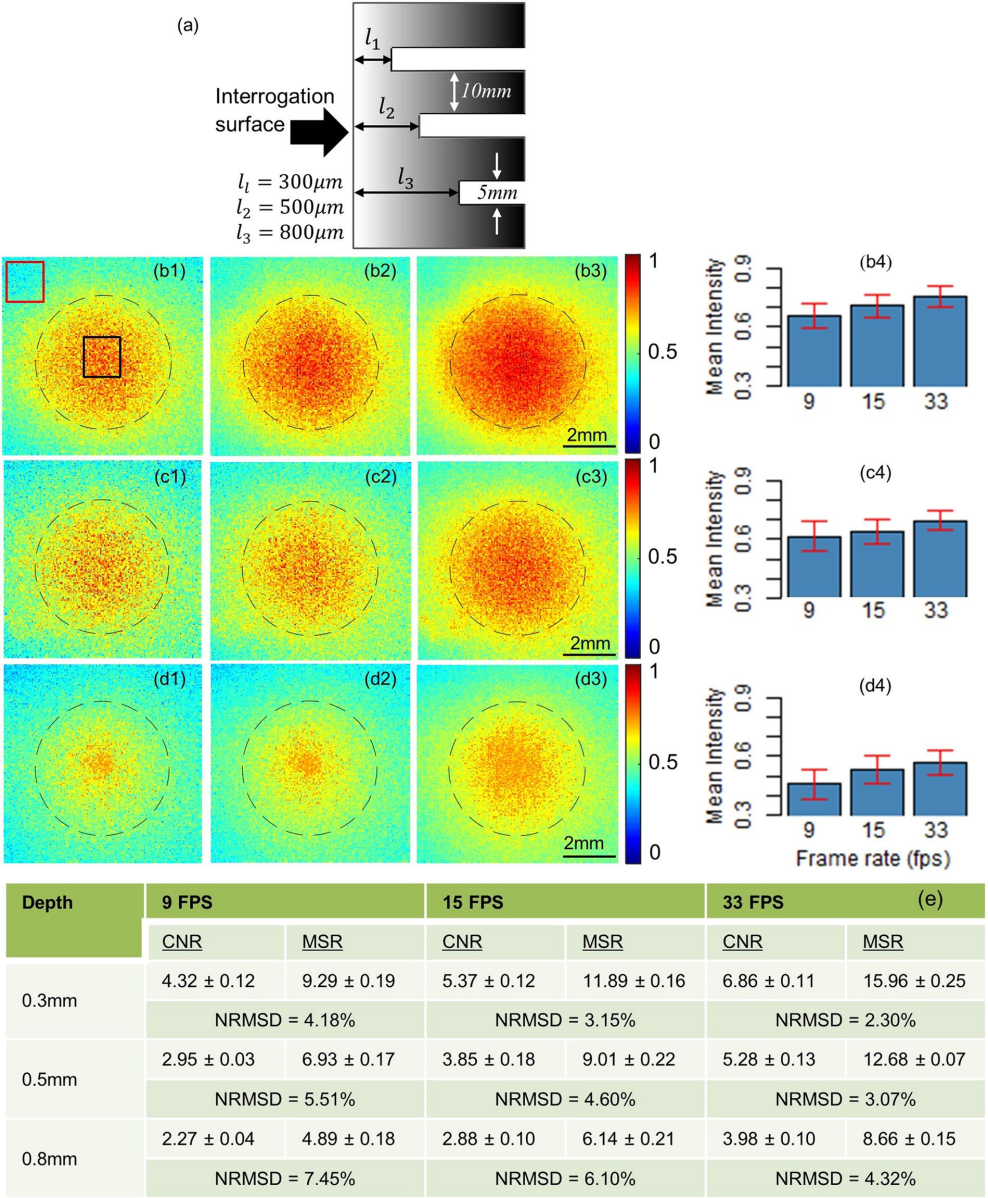
(**a**) A schematic diagram of the cross-section of the block with a blind hole at 300 µm, 500 µm, and 800 µm beneath the interrogated surface. (**b**1–**b**3) Amplitude images from 300 µm hole obtained from LIT demodulation at camera frame rates of 9 fps, 15 fps and 33 fps, respectively. (**c**1–**c**3) represent similar images from 500 µm hole and (**d**1–**d**3) from 800 µm hole. The dotted circle in each image represent the true size of the hole. Mean normalized intensity of the pixels inside the hole at different frame rates from 300 µm (**b**4), 500 µm (**c**4), and 800 µm (**d**4) holes. The error bar indicates the STD of the pixels inside the hole. Table (**e**) depicts mean ± STD of MSRs and CNRs and the averaged NRMSD. The STDs in MSR and CNR were calculated from the 3 repeated measurements on the same hole at the same frame rate. MSRs and CNRs were calculated from the background (red rectangle in (**b**1)) and the foreground regions (black rectangle in (**b**1)) from all images.

Another key advantage of higher frame rates in LIT is improvement in signals’ SNR that eventually translates to an improvement in LIT images quality and reliability. To demonstrate this concept experimentally, we carried out LIT experiments with the developed system at frame rates of 9, 15 and 33 fps, corresponding to the nominal frame rate of Seek thermal camera, results published in our previous work^29^ and the current imaging system, respectively. In these experiments, the sample was an Aluminium block (45 mm × 25 mm × 30 mm) that had three drilled subsurface holes of the diameter of 5 mm, simulating circular defects 300 µm, 500 µm and 800 µm below the intact interrogation surface, as shown schematically in Fig. 3a. The laser beam was intensity-modulated at 1 Hz and covered a circular area on the sample surface with a diameter of 2 cm. To test the repeatability, experiments were repeated 3 times for each subsurface hole at each frame rate and average normalized root-mean-square deviation (NRMSD: equation (2); Methods section) was calculated. The NRMSD is one commonly used quantitative measure of the deviations of repeated measurements from the mean value. In Fig. 3, panels b1–b3 depict the LIT amplitude images obtained from the shallowest defect (300 µm) at camera frame rates of 9, 15 and 33 fps, respectively. Similarly, panels c1–c3 and panels d1–d3 show images of defects 500 µm and 800 µm below surface, respectively. The dashed circle in all images indicates the true location and size of the subsurface holes. In these images, the defective area (hole) can be realized in amplitude images as a region of higher amplitude, confirming additional diffusive contributions of thermal energy to sample surface as a result of the introduction of thermal impedance by the defect. These observations are consistent with those obtained in our previous benchmark tests using expensive research-grade IR cameras^7,30,31^. While defects can be detected in all 3 frame rates, the defect contrast, and consequently reliability of defect detection, is directly correlated with an increase in the frame rate. The true size of the holes is better recognized in the amplitude image obtained at higher frame rates. To quantitatively compare the images at different frame rates, pixels inside the circles were extracted and analyzed. Panels b4, c4, and d4 depict the mean and standard deviation (STD) of pixels inside circular defective regions for the 300 µm, 500 µm and 800 µm subsurface holes, respectively. For a given hole, the mean intensity increases, while the STD decreases, as the frame rate increases. However, the mean values were not significantly different among different frame rates for a given hole (Paired t-test p > 0.05, n = 3 repeated experiments for each hole). Nevertheless, the effect of frame rate on image quality metrics was significant: contrast-to-noise ratio (CNR) and mean-to-standard-deviation ratio (MSR) were calculated from the amplitude images: MSR measures the smoothness of regions and is calculated from the regions that have a homogeneous appearance. The CNR, on the other hand, measures the contrast between the foreground and background regions, representing the ability to visualize the defect in the image through the noise. The table in panel (e) depicts the MSRs and CNRs values computed from the amplitude images at 3 different frame rates. The STDs were calculated from 3 repeated experiments in each hole at each frame rate. This table demonstrates a considerable improvement of both MSR and CNR by increasing the LIT system camera frame rate. The paired t-test shows that CNR is significantly increased (p < 0.05) with the increased frame rate in all the pairwise comparisons, except 15 fps vs. 33 fps and 9 fps vs. 15 fps with 500 µm hole and 15 fps vs. 33 fps with 800 µm hole. Similarly, MSR significantly increased (p < 0.05) with the increase in frame rate in all the pairwise comparisons, except 9fps vs. 15 fps with hole 500 µm and 800 µm. These statistical analyses indicate that LIT with developed SDK at 33fps results in significantly better MSR and CNR compared to those obtained at native camera frame rate (i.e., 9fps). Moreover, the average NRMSD decreases with increasing the frame rate indicating better repeatability with the higher frame rate. The averaged NRMSD, at a given frame rate, increases with increasing depth of the defect due to the degradation of signals’ SNR from deeper holes as a result of the damped and diffuse nature of thermal waves. The histograms of the amplitude image values from the defect and background regions at camera frame rates of 9, 15 and 33 fps for the three holes are included in Supplementary Fig. 1. These histograms, also, demonstrate the reduction of distributions’ STD (Supplementary Table 1) with the increase in frame rate which, from a statistical point of view, translates to improvement in the reliability of defect detection.

The results of aluminum samples demonstrate the importance and significance of having higher frame rates in LIT, especially in a low-cost system that is prone to noise. Moreover, an increase of frame rate in LIT platforms extends the range of applications of LIT systems by enabling proper sampling of higher frequency thermal waves that are required for interrogation of thinner samples (e.g., coatings) and/or smaller defects in accordance to the concept of thermal-diffusion length. It should, however, be noted that conducting AT at higher frame rates results in acquisition of more data and require more computing power, which can become problematic if data analyses are to be carried out by personal portable devices like cellphones. Utilizing real-time lock-in processing algorithms instead of fast Fourier transform or performing data analyses with more powerful tablets are few approaches for overcoming this potential limitation.

To demonstrate the potential impact of the development of reliable, yet low-cost, LIT systems, sections below depict experimental results obtained by our developed system in high impact areas of detection of human early dental caries and detection and quantification of cannabis consumption from oral fluids.

### Low-cost LIT system for the detection of early dental caries

Dental caries remains the most prevalent chronic disease in both children and adults worldwide^32–34^. Detection of dental caries at early stages is of prime importance in Dentistry as the progression of caries can be stopped (or even reversed) only at early stages^35,36^. However, existing clinical methods in Dentistry (X-ray and Visual/Tactile Inspection) do not have sufficient sensitivity to detect early stages of tooth demineralization^37^. Our research results with research-grade LIT systems^18,19^ demonstrate the possibility of detecting caries at early stages, but the cost and size of LIT systems have always posed a barrier to translation of this technology to Dentistry. To demonstrate the possibility of performing detection of early caries using a low-cost and a small size LIT system, we carried out detection experiments on extracted human teeth with artificially induced early caries. A demineralization gel was prepared (detailed in method section) to induce caries in a controlled manner on healthy extracted human teeth. This gel mimics the properties of bacterial plaques on enamel, providing an environment for the cyclic occurrence of demineralization and remineralization and thus the creation of early caries^30^. For the sample reported in this manuscript, two treatment windows were created on the tooth by exposing the left and right treatment windows to the gel for 3 and 7 days, respectively.

**Figure 4:**
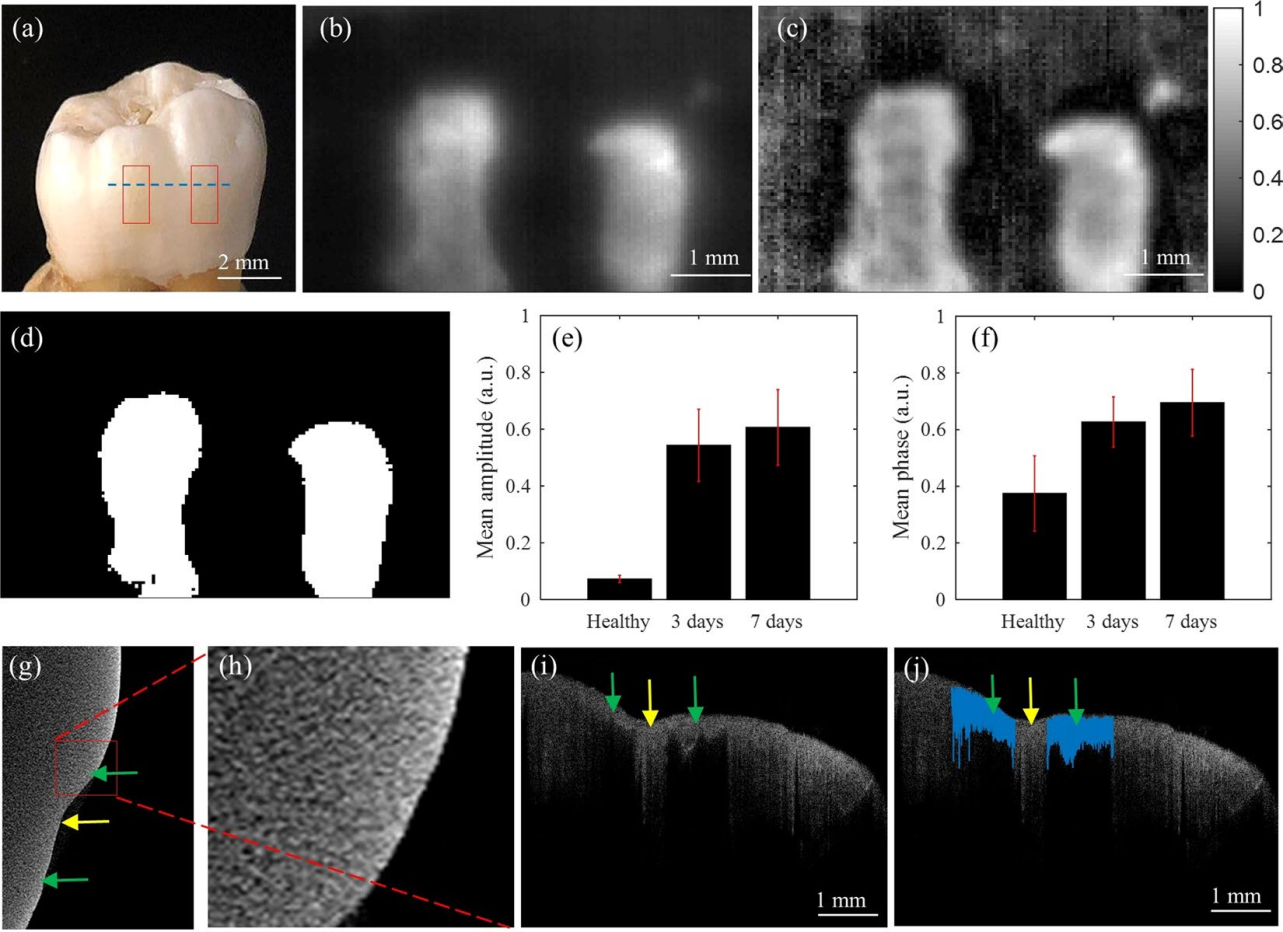
(**a**) A visual photograph of a dental sample; left and right treatment windows were demineralized for 3 and 7 days, respectively. LIT (**b**) Amplitude and (**c**) phase images obtained at 2 Hz modulation frequency. (**d**) segmented demineralized windows. (**e**) Mean amplitude and STD from the healthy and demineralized areas shown in (**d**). (**f**) Mean phase and STD from the healthy and demineralized area shown in (**d**). (**g**) μCT slice taken from treatment windows (green arrows). (**h**) Zoomed area of a treatment window (**i**) OCT B-scan taken from the treatment windows along the dashed line in (**a**). (**j**) Segmented demineralization areas in blue; average OCT penetration depths: 180 µm for 3 days and 163 µm for 7 days; average OCT intensities: 49.52 ± 27.90 dB for 3 days and 54.68 ± 29.50 dB for 7 days.

The visual photograph of the sample after artificial demineralization is shown in Fig. 4(a); red rectangles depict the locations of the two treatment windows. The size of the demineralization windows was approximately equal to 1mmx2mm and the distance between two windows was ~1.5 mm. While visual inspection of the sample after demineralization could not detect any colorimetric abnormalities/white spot lesions, the presence of demineralization is clearly detected by the developed low-cost LIT system in both amplitude and phase images obtained at a modulation frequency of 2 Hz, Fig. 4(b,c). In the amplitude image, Fig. 4(b), a higher intensity of demineralized region is due to the enhancement in local absorption of laser illumination by demineralization by-products; thus, generating a thermal wave of higher amplitude compared to the surrounding intact areas^38^. The treatment windows are seen as areas of different contrast in the phase image as well, Fig. 4(c). This is because the enhancement of light absorption in caries region shifts the local centroid of the thermal wave field, causing a phase shift in the thermal signals compared to those of the surrounding intact areas.

In order to quantify the diagnostic performances of our low-cost LIT system for detecting early dental caries, the contrast value of pixels inside the treated windows was compared to those of the healthy region. Figure 4(d) shows the segmented healthy, 3 days demineralization and 7 days demineralization areas. The pixel values of the treated windows were considerably higher than the average pixel value of the whole image; therefore, they can be reliably segmented from the intact surfaces by simple thresholding. After thresholding, means and STDs of the pixels inside the healthy and treated windows were calculated and compared. Figure 4(e,f) show the average intensity of the healthy, 3 days demineralization and 7 days demineralization windows from the amplitude and phase image, respectively. The error bars on bar plots indicate STD. The average intensities of the treatment windows are considerably higher than that of the healthy region. There was no overlap between the error bars of healthy and 3 days demineralization window in both amplitudes and phase images, indicating a difference between the mean values. Although the error bars of 3 days and 7 days treated windows are overlapped, the mean amplitude and phase values of 7 days demineralization window are higher than those of 3 days demineralization window suggesting the presence of more advanced caries in the 7-day demineralization window. The results of the study are consistent with our previous LIT studies of early dental caries with high-end research-grade IR cameras^18,19^ that also show increased contrast in the amplitude and phase images at caries sites compared to the healthy areas.

Figure 4(g) depicts a Micro-Computed Tomography (μCT) slice of the sample taken along the dashed line indicated in Fig. 4(a). Due to the small extent of demineralization and inferior sensitivity of X-ray to demineralization, μCT is unable to detect the treatment window in both 3 days and 7 days of the demineralization periods. Figure 4(h) is a magnified μCT image of a treatment window that is unable to show the material loss due to demineralization. Figure 4(i) shows the OCT B-scan taken from the healthy and treatment windows along the dashed line indicated in Fig. 4(a). Here, the two treatment windows are clearly visible due to significant enhancement of light scattering at early caries sites and shadowing of deeper regions. The treatment windows in the OCT images are segmented, blue color in Fig. 4(j), and the average OCT penetration depth and intensity are calculated. OCT results suggest reasonably similar penetration depth for the two treatment windows (180 µm for 3 days and 163 µm for 7 days). Slightly less penetration depth of the 7-day treated window is due to the increase in light scattering due to additional demineralization compared to the 3-day treated window. The averaged OCT intensities of the two windows were also similar (49.52 ± 27.90 dB for 3 days and 54.68 ± 29.50 dB for 7 days). The results of this study support previous findings that the optical scattering increases with mineral loss in artificially demineralized dental enamel^36^. The results of Fig. 4 demonstrate the ability of developed LIT system for detecting early stages of demineralization, which is at least 2 orders of magnitude lower in cost compared to the competing early caries detection technology of OCT.

### Low-cost LIT system for the detection and quantification of THC in oral fluid

With recent changes in the legalization of cannabis around the world, there is an urgent need for rapid, yet sensitive, screening devices for testing drivers and employees under the influence of cannabis at roadside and workplace, respectively. To determine the viability of our technology for detecting THC (the principal psychoactive constituent of cannabis) at point-of-care, we conducted experiments with the commercially available saliva-based lateral flow immunoassay (LFA) test strips. A detailed description of LFA technology can be found elsewhere;^39^ but, briefly, LFAs are simple paper-based devices used for the detection and quantification of analytes in a complex mixture. A developed LFA test strip shows two colored bands: the test and the control bands. The intensity of color at the test band correlates with the amount of target analyte (e.g., THC) present in the solution, while the presence of the control line ensures the validity of the experiment. In this LIT study, commercially available oral fluid LFA strips for detection of THC with a nominal detection limit of 25 ng/ml were used. These LFAs were manufactured in a competitive format, in which an increase in THC concentration yield reduction of color intensity in the test band.

**Figure 5:**
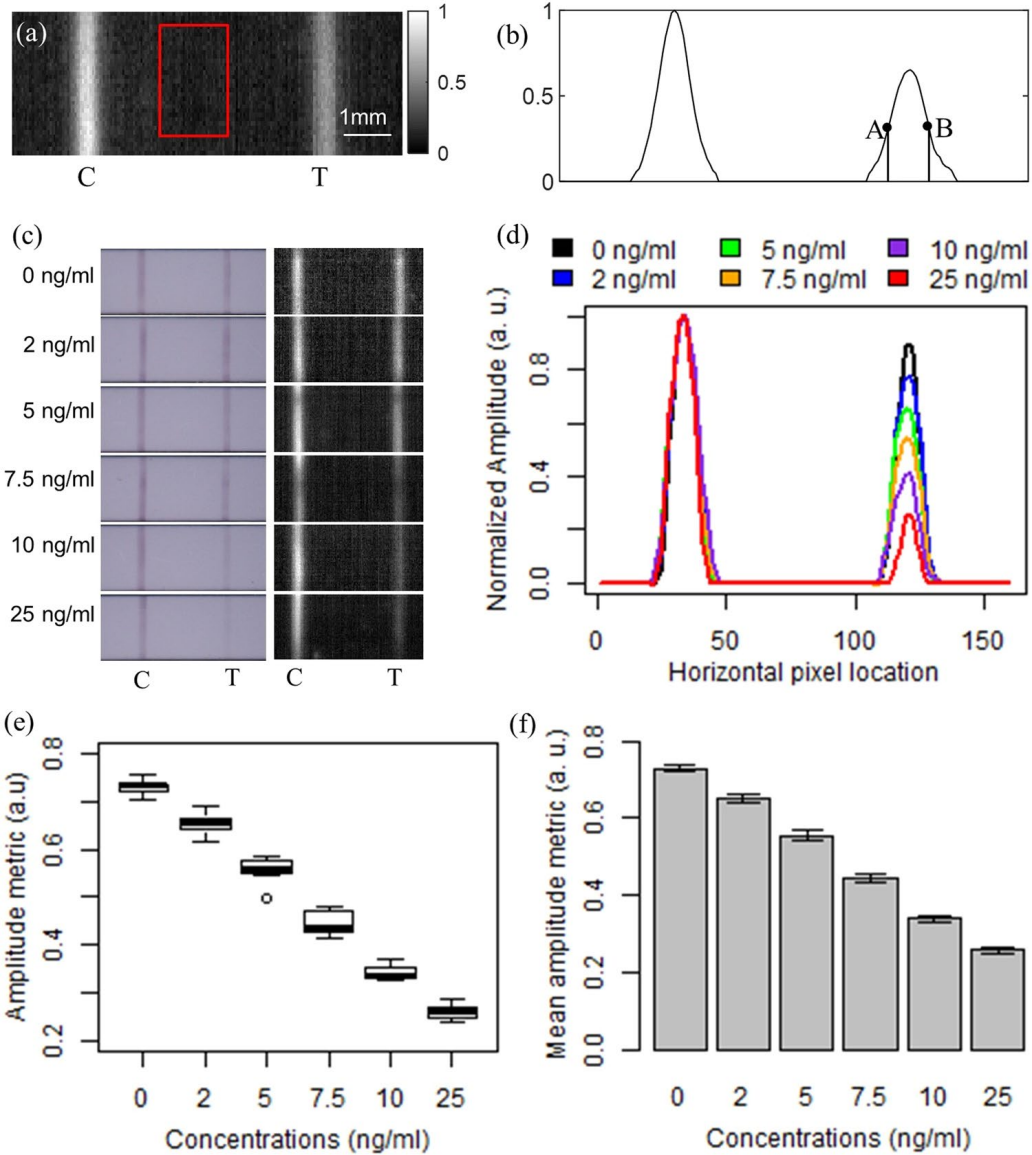
(**a**) Representative LIT amplitude image of LFA spiked with 5 ng/ml THC-saliva solution. (**b**) Two curves obtained at the control and test line by averaging the pixels in the vertical direction of (**a**). (**c**) Visual microscopy and LIT comparisons of representative LFAs at different concentrations. (**d**) Comparison of amplitude metric between panel (**c**) 6 representative LFAs at concentrations 0, 2, 5, 7.5, 10 and 25 ng/ml. (**e**) distribution of amplitude metric from all the LFAs at different concentrations (n = 240). (**f**) Comparison of mean amplitude metric among different concentrations. The error bar shows the 99% confidence interval.

These LFAs were spiked at six different concentrations: 0, 2, 5, 7.5, 10 and 25 ng/ml. Eight LFAs were spiked at each concentration. LIT experiments on LFAs were performed at laser modulation frequencies of 2 Hz. To test the repeatability, each LFA was imaged 5 times. Figure 5(a) shows a representative amplitude image of LFA strip spiked at 5 ng/ml of THC in oral fluid. The left and right lines depict the control and test band, respectively. These bands are detected as areas of higher thermal wave amplitude because the immobilized gold nanoparticles (GNPs) at these sites efficiently absorb the laser excitation through surface plasmon resonance. To minimize the systematic errors induced by day-to-day variations in laser illumination system and manufacturing of LFAs, the contrast of LFA test strips in amplitude image were normalized with respect to those of the surrounding white nitrocellulose paper (red rectangle shown in Fig. 5a). For quantitative analysis, the average intensity of pixels over all the rows in a strip was calculated. The aforementioned process yields a plot with two bell-shaped curves, representing the control and test bands as seen in Fig. 5(b). A metric was defined by averaging the amplitude values within the full width at half maximum (FWHM) of the test band (between points A and B in Fig. 5b). Throughout this paper, we will adopt this value as the amplitude metric. All quantitative analyses were carried out using this metric.

Figure 5(c) shows visual microscopy and LIT amplitude images of 6 representative LFAs at various THC concentrations. It can be seen that the decrease in the contrast at the test lines is associated with the increase in THC concentration in both visual and LIT images. However, the change of test line contrast with a decrease in THC concentration is more pronounced in LIT images, suggesting better detection sensitivity of LIT compared to visual interpretation. Figure 5(d) shows the amplitude metrics obtained from the six LFAs shown in Fig. 5(c); the peak values of the test line curves show clear correlation with the concentration of THC. To verify enhanced detection performance of LIT over visual observation, the amplitude metrics were calculated for all LFA measurements. Figure 5(e) depicts the distribution of all the amplitude metrics (n = 240: six concentrations × eight LFAs at each concentration × each LFA imaged five times). Each box displays the amplitude metric values of the LFAs of the same concentration (n = 40: eight LFAs at each concentration × each LFA imaged five times). The box plot shows that there is no overlap in the distribution of data between any two concentration groups. The mean amplitude metrics with 99% confidence interval error bars are depicted in Fig. 5(f), showing no overlap between the error bars of any two concentration groups. To compare mean amplitude metric values among different THC concentration groups, the one-way analysis of variance (ANOVA) was conducted. One-way ANOVA test showed statistically significant differences between the mean normalized amplitude values of different concentration groups (p < 0.001). Tukey’s honestly significant difference (Tukey’s HSD) test was adopted to perform the pair-wise comparison. Tukey’s pairwise comparisons showed that all the pairwise group comparisons were significantly different (p < 0.001). Effect size of 0.97 and statistical power of 1.0 was obtained at a significance level of 0.01 and a sample size of 40 in each concentration group. The post-hoc pair-wise comparison illustrates that the developed low-cost LIT system can reliably differentiate LFAs spiked at 2 ng/ml concentrations from the 0 ng/ml, demonstrating suitability for enforcement of the 2 ng/ml legal *per se* limit in many jurisdictions. Sensitivity, specificity, and accuracy of differentiating THC concentrations of 2 ng/ml and more from 0 ng/ml were found to be 95% that is considerably higher than the >80% standard set by Driving Under Influence of Drugs, Alcohol and Medicines (DRUID) for the nominal detection threshold of a drug screening device^40^. The data of Fig. 5(f) can also be seen as calibration data through which THC concentration in saliva samples can be quantified.

The LFAs used in this study are low-cost solutions that are designed by the manufacturer to be interpreted visually. We recently conducted a human study to determine the limit of detection of visual interpretation of these LFAs; we also compared interpretations of human vision to those from a research-grade benchtop LIT system that incorporated an expensive (~$10k) infrared camera^41^. The results of that study revealed detection limits of 10 ng/ml and 2 ng/ml for interpretation by human vision and research-grade benchtop LIT system, respectively. The results of Fig. 5, as such, demonstrate that the low-cost LIT system described in this manuscript provides comparable performance to that of high-end research grade LIT systems at fraction of the cost, paving the way for commercialization and widespread adoption of this AT technology.

The size (~15 cm × 10 cm × 8 cm) and cost (~$800) of our system are less than those of commercially-available oral fluid DRAGGER DRUG TEST 5000. Therefore, it has great potential for translation to roadside and workplace as a low-cost and portable testing device, while offering THC detection performance that aligns with DRUID expectations. In addition, the portable thermo-photonic reader is expected to offer lower consumable cost compared to the existing solutions as it utilizes commercially-available inexpensive (~$8) colloidal gold nanoparticle-based LFAs.

## Conclusions

AT is a widely used non-destructive testing technique for the detection of defects based on their radiometric thermal signatures. To date, AT has been utilized for interrogation of a broad spectrum of materials, spanning from the detection of manufacturing defects in industrial parts to the detection of diseases in biological specimens. However, despite the wide span of applications, the commercialization of AT technologies has long been impeded by the high cost and large size of the IR cameras used in this technology. In this manuscript, we report on the possibility of performing scientific and reliable AT using low-cost and size cellphone attachment IR cameras. Our results suggest that the developed SDK not only allows for on-demand control of camera attributes but also enables reliable and consistent acquisition of IR images at a high frame rate of 33 fps from a ~$250 camera with a nominal frame rate of 9 fps. To demonstrate the impact of developed low-cost LIT systems, we present and discuss two high impact biomedical applications. Our results on the detection of early demineralization of human tooth clearly demonstrate the ability of the developed low-cost system in detecting early caries; thus, paving the way for translation of AT technologies to Dentistry as a preventive tool for alleviating the significant financial and societal burdens of dental caries on families, governments, and health care systems. Our demonstration of the possibility of measuring THC in oral fluid with the low-cost system, enables commercialization of a low-cost point-of-need portable device, for roadside or workplace, for detecting and quantifying THC at an unprecedented low detection threshold of 2 ng/ml; a task not achievable by any commercially available point-of-need devices in the market.

## Methods

### Principle of Lock-in Thermography (LIT)

The principle of LIT is based on the introduction of a periodically modulated light source on the surface of the sample and monitoring the resulting local surface temperature of the sample via an IR camera^4^. Absorption of modulated light excitation creates a modulated temperature field (aka thermal-wave field) inside the sample extending axially to a depth proportional to the thermal diffusion length^42^. In this configuration, the presence of defects alters the centroid of the induced thermal-wave field in the defective region, resulting in depth-integrated radiometric signals different from those registered in intact areas. The lock-in demodulation of the depth-integrated signals leads to the calculation of phase and amplitude images^6^. Since the intensity-modulation of excitation in LIT is carried out at a single frequency (*f*_*m*_), the LIT demodulation of temporal radiometric responses reveals the defective areas in both amplitude and phase images/channels^43^. The presence of subsurface defects, normally, results in an increase in amplitude values as the thermal impedance introduced by defects results in an additional diffusive thermal contribution to sample surface temperature. Defects also can be identified in phase images because of the alteration of the center of gravity of the thermal-wave field in the defective zone by the introduction of thermal impedance. While generally, amplitude images offer better SNR, LIT phase images are inherently emissivity-normalized and as such not prone to errors caused by variations in emissivity of sample surface^44^. Another key advantage of LIT is its depth profilometric nature. That is, inspection depth in LIT is controlled via the intensity-modulation frequency of external excitation, ${f}_{m}$, and in accordance with the definition of thermal diffusion length (i.e., $\mu =\sqrt{\alpha /\pi {f}_{m}}$ with $\alpha$ as thermal diffusivity). As a result, probing deeper into the sample can be achieved by reducing the modulation frequency, while LIT inspection at high modulation frequencies is suitable for the detection of superficial defects (e.g., coatings defects).

#### Development of the low-cost lock-in thermography system

**Figure 6:**
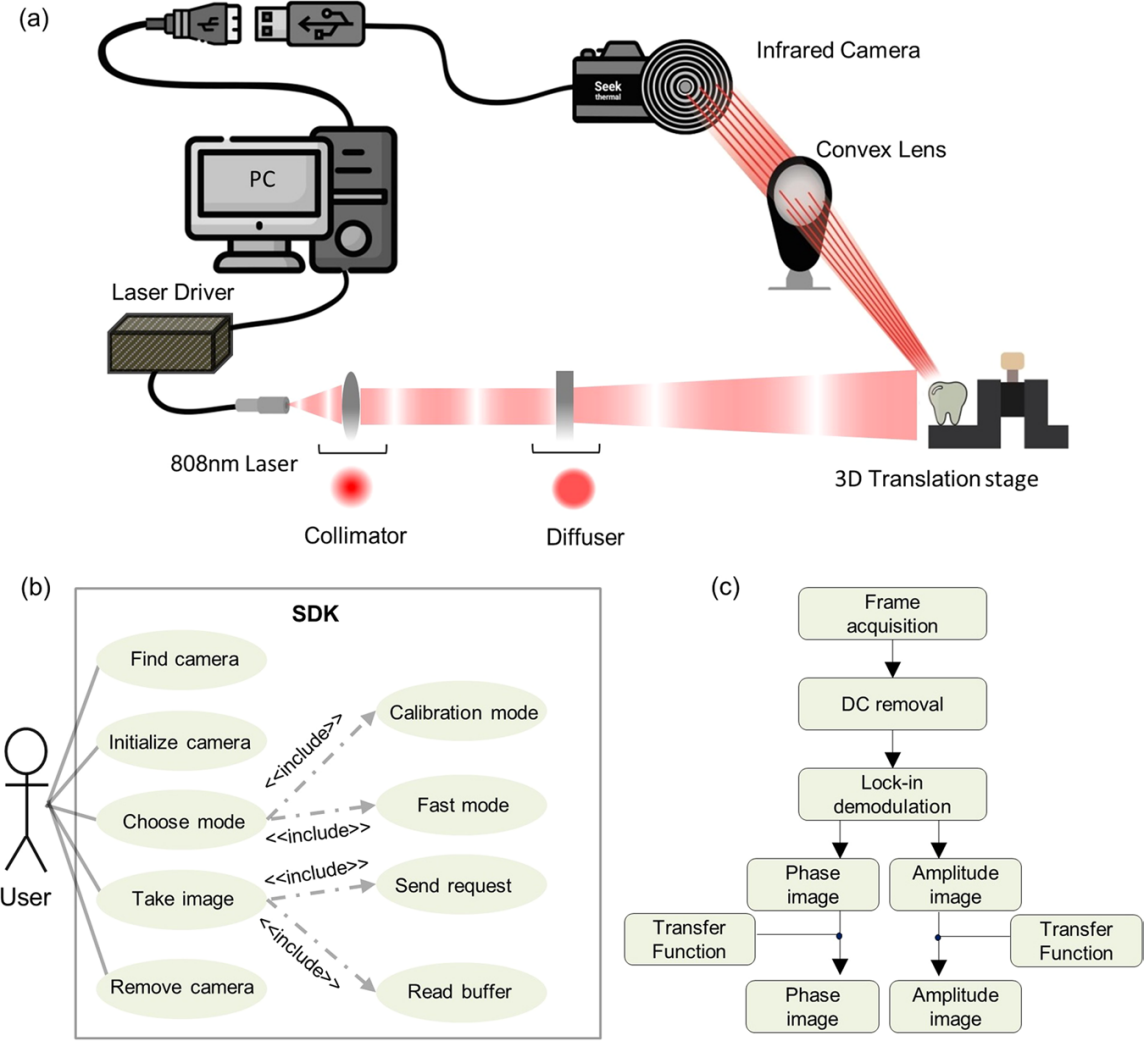
(**a**) Schematic diagram of the lock-in thermography system (**b**) A use case diagram that depicts a blueprint of the functionalities provided by our developed SDK. (**c**) A flow chart showing the signal processing method applied to the waveform at each pixel of the image.

A schematic diagram of the LIT imaging system built in our lab using a low-cost cellphone attachment IR camera is shown in Fig. 6a. A fiber-coupled (core diameter = 200 um), continuous-wave near-infrared laser with a center wavelength of 808 nm (Jenoptik, Jena, Germany) was used as an excitation light source. The intensity of excitation light was collimated and homogenized using a collimator and optical diffuser (Thorlabs, Newton, New Jersey, USA, F220SMA-780 and ED1-C20-MD). The sample was secured on a LEGO-jig and mounted on a 3-axis translation stage and excited by the light source. A multifunctional data acquisition unit (National Instruments, Austin, Texas, NI USB-6363 BNC) was used to modulate laser intensity at the desired modulation frequency ($${f}_{m}$$). The thermal responses of the sample were registered by using a low-cost cellphone attachment IR camera (SEEK THERMAL COMPACT; Seek Thermal Inc.; Android; 156 × 207 Pixels) in conjunction with a low-cost (~$10) CO2 laser cutting Zinc Selenide objective lens (f = 1′′).

While the nominal frame rate of the camera through its standard applet is less than 9 fps, we have managed to utilize USB 2.0 documentation and Microsoft Windows native application programming interfaces (APIs, such as WinUSB and SetupAPI) in order to set up packets of information and send them to the camera’s default endpoint address and, subsequently, acquire frame data from the camera through a corresponding pipe. As such, the developed platform has not only the ability to control camera attributes through a simple USB interface but also can achieve a stable high frame rate of 33 fps through a circular buffer hierarchy and multi-threading. In addition, the program controls the camera shutter and calibrate the camera before capturing the frames, providing stable frames up to a maximum frame rate of 33 fps after calibration. In order to compensate for the non-uniform sampling of data, in our previous work^29^ we recorded timing information of each frame captured by the camera and employed spline interpolation at a uniform sampling frequency before applying LIT demodulation. The developed SDK, on the other hand, has the ability to capture frames at a constant frame rate, so interpolation is not necessary before the LIT demodulation of thermal signals.

Figure 6b is a use case diagram that depicts a blueprint of the functionalities provided by our developed SDK for controlling the camera and capturing camera frames at a constant and uniform frame rate. First, the user initiates the SDK and checks if the IR camera is connected to the system. Then, the user adjusts the camera setting and initializes the camera for display and visualization of raw frames. Once these initialization processes are carried out, the SKD allows the system to take raw frames in two modes: calibration mode and fast mode. In the calibration mode, camera frames are captured while the shutter is closed and the camera’s internal non-uniformity correction map is recalibrated. The user can adjust the number of frames required for the calibration of the camera. Once the camera calibration is done, the system is set to take images in fast mode at the desired camera frame rate up to a maximum of 33 fps and without any disruption in the acquisition. Capturing frames in fast mode are carried out by sending a request for a frame acquisition followed by a read buffer command to move the acquired frame and time stamp information from camera memory. Once all images are captured, the camera is removed from the system and these captured frames are processed for LIT demodulation as described in Fig. 6c.

Figure 6c depicts the signal processing steps applied to the temporal signal of each camera pixel for LIT demodulation. That is, after acquiring frames, first the effect of bulk heating is removed from the temporal signal by fitting a polynomial curve of order seven. Then, a fast Fourier transform (FFT) is applied to the waveform, followed by determination of the magnitude and phase of the complex number corresponding to the laser modulation frequency in the Fourier domain. At last, the transfer function of the system is applied to the amplitude and phase images to correct imperfections caused by nonuniformities in the illumination and collection optical sub-systems. The transfer function was calculated by performing lock-in imaging on a thick Aluminum block painted with matt black paint on the front surface that resembles a semi-infinite blackbody sample. The theoretical amplitude and phase responses of such samples are known. Therefore, we found the experimental amplitude and phase images at desired modulation frequencies and then calculated the compensation factor for each camera pixel that once applied to the experimental pixel value, yield the theoretical prediction. With this practice, any systematic error caused by illumination and/or acquisition sub-systems can be compensated for. Once the amplitude and phase transfer functions at given modulation frequency are calculated based on the semi-infinite sample, they can directly be applied to amplitude and phase images of any sample to remove the effects of system systematic errors.

#### Spectral-domain optical coherence tomography

A spectral-domain OCT system that comprised of a 1315 nm near-infrared superluminescent diode (Exalos; Switzerland) light source (maximum power 30 mW) was used to take images of demineralized tooth. The OCT system is based on Michelson interferometry in which 50/50 of light is split into the reference and sample arms by a fiber coupler. A polarization controller is used in the reference arm to adjust polarization to the cross-polarization state. A-line scan camera in the spectrometer was composed of 2048 pixels and could acquire A-lines up to a maximum scan/acquisition rate of 140 kHz. The theoretical axial and lateral resolution of the system in teeth is approximately 5 μm and 10 μm, respectively. A GPU (Graphics Processing Unit) based processing was developed for real-time display of OCT B-scans (i.e., cross-sectional image). The B-scan captured from the system were used for studying the effects of demineralization. To find the depth of caries using OCT images, the location of the surface where the scattering occurs and the location of the surface where the scattering ends is thresholded in each vertical line inside the demineralization windows. The color-coded areas in Fig. 3j indicate the thresholded area. Since the depth of each vertical line is unequal, average depth is reported as the depth of caries.

#### Micro-computed tomography

Micro-Computed Tomography (SKYSCAN 1272 high-resolution µCT system, Bruker MicroCT, Kontich, Belgium) was used to take images of the demineralized teeth. The sample was placed in a LEGO block such that the treatment window would fall in the field of view of the µCT detector. A 0.5-mm Al/0.038-mm Cu filter was used to minimize the effects of beam hardening. The tooth was scanned at a rate of 87 kV with camera pixel size of 7.4 µm and exposure time of 2000 ms per frame and a rotation angle of 0.1 degrees. The µCT images (n = 2813) were reconstructed from 1920 projections using NRECON software (Version 1.7.1.6., Skyscan, Kontich, Belgium).

#### Aluminum samples

To examine acquisition disruptions and frame rate of manufacturer’s applet and developed SDK (Fig. 1) LIT experiments were carried out on thick Aluminum block (40 mm × 30 mm × 30 mm) with no internal defects. To test the advantage of a higher frame rate for better spatial resolution, an aluminum pin fin thermal heat sink sample was used (Fig. 2a; Wakefield-Vette, New Hampshire, United States). The dimension of the heat sink is 40.6 mm × 40.6 × 13.3 mm and consists of pin fins of cross-section size 1.4 mm × 2.4 mm beneath the 2-mm thick plate, as shown in Fig. 2a. LIT experiments were conducted on plate surface of the thermal heat sink (area shown by the red dashed rectangle in Fig. 2a). The area imaged on the 156 × 207 pixels of the camera was 7.43 × 9.86 mm^2^, respectively. The spatial pixel size of images from the interrogated surface is, therefore, ~48 µm. Raw frames were collected for 90 seconds and those frames were demodulated according to lock-in principles to compute amplitude images. To study the subsurface defect (Fig. 3a), a custom-made Aluminium block (dimensions 45 mm × 25 mm × 30 mm) was used. Three subsurface circular holes of diameter 5 mm were created by drilling the block from the back side. The aforementioned simulates circular defects at 300 µm, 500 µm, and 800 µm below the intact interrogation surface. The thermal diffusivity and conductivity of the aluminum were 9.1 × 10^−5^ m^2^/s^45^and 205 W/mK^46^, respectively. In all LIT experiments carried out on aluminium samples the average optical intensity on the sample surface was 1.6 W/cm^2^ and temporal temperature responses were recorded for 60 seconds.

#### Dental sample

The anonymous human teeth were collected from local oral surgeons and in accordance to the bio- and laser safety guidelines in place at York University. These specimens were stored in distilled water inside the fume hood to prevent dehydration. In order to induce demineralization in specimens in a controlled manner, an acidified gel was prepared by mixing 0.100 M lactic acid and 0.100 M sodium hydroxide to give a pH value of 4.5 and then adding 6% w/v hydroxyethyl cellulose^18,19^.

A tooth with no visible defect or white spot lesion was selected, rinsed thoroughly with distilled water and dried in the air before exposure to the demineralizing gel. In order to achieve localized demineralization, the surface of the tooth was covered by transparent nail polish, leaving small windows (aka treatment windows) exposed. The sample was then submerged upside down in a test tube containing 25 ml of acidified gel for specified durations of demineralization. Two treatment windows were created on the tooth by exposing the left and right treatment windows to the gel for 3 and 7 days, respectively. At the conclusion of artificial demineralization, the sample was removed from the test tube, rinsed under running water, nail polish was removed using Acetone and rinsed again with water before conducting LIT imaging. To take LIT images of the dental sample, the cellphone attachment camera was focused on the surface of the sample that is securely mounted on a LEGO block. LIT imaging was carried out on dental sample by recording the thermal responses for 30 seconds at modulation frequency of 2 Hz. The average optical intensity on the sample surface was 1 W/cm^2^.

OCT imaging was conducted at scan/acquisition rate of 100 KHz. Polarization controller in reference arm was adjusted to the cross-polarization state to minimize surface reflections. To segment the demineralized areas from the healthy area in B-mode images, mean and STD of the amplitude image was calculated and a thresholding operation was applied to the entire image. The global threshold value was calculated by computing the average pixel plus one STD of all the pixels of the image.

#### Preparation of oral fluid THC solution and LFA strips

To spike LFAs at different THC concentration, saliva samples containing Δ^9^-THC was prepared by adding known volumes of Δ^9^-THC stock solution (MilliporeSigma; Oakville, Canada) to non-stabilized artificial saliva (Pickering Laboratories, Inc, Mountain View, California, USA). The THC solution was prepared following the standardized procedure suggested by the Canadian Society of Forensic Science Drugs and Driving Committee was followed^47^. Commercially available oral fluid LFA strips (NARCOCHECK Saliva Test Strips, Kappa City Biotech SAS, Montluçon, France) were used. Six different concentrations (25, 10, 7.5, 5, 2 and 0 ng/ml) of THC-saliva solution was prepared. In each experiment, 150 µL of the solution was pipetted to the sample pad of the LFA strip. Eight LFAs were spiked at each concentration to investigate reproducibility and each LFA was interrogated five times using the developed low-cost LIT system to investigate repeatability. All LIT experiments were carried out with average optical intensity of 1.6 W/cm^2^ and temporal temperature responses were recorded for 60 seconds.

### Data analysis

The SNRs of waveforms from our developed SDK and manufactures applet was calculated from the time signals spectra using the following Fourier-domain definition:

1 $$SNR=20\,{\log }_{10}({A}_{s}/{A}_{n})$$

here, *A*_*s*_ is the amplitude of the signal at the applied modulation frequency of 1 Hz and *A*_*n*_ is the average amplitude of the noise in the region depicted in Fig. 1c,d. For the calculation of SNR, an average amplitude of noise was calculated from the frequency range of 0.2 Hz to 0.8 Hz, (regions shown by the red dashed rectangles in Fig. 1c,d).

The root-mean-square deviation (RMSD) and the normalized RMSD are defined as^48^

2 $$RMSD=\sqrt{\frac{{\sum }_{i=1}^{n}{({x}_{i}-{\bar{x}}_{i})}^{2}}{{\rm{n}}}}\,\& \,NRMSD=\frac{RMSD}{{\bar{x}}_{i}}$$

where *x*_*i*_ is the intensity of a pixel in an experiment and ${\bar{x}}_{i}$ is the mean intensity of n repeated experiments for i^th^ pixel. The NRMSD was calculated for each LIT image at each frame rate.

MSR and CNR were calculated using following definition^49^:

3 $$CNR=\frac{|\overline{{\mu }_{f}}-\overline{{\mu }_{b}}|}{\sqrt{0.5({\sigma }_{f}^{2}+{\sigma }_{b}^{2})}}\,\& \,MSR=\frac{\overline{{\mu }_{f}}}{{\sigma }_{f}}$$

where $\overline{{\mu }_{f}}$ and ${\sigma }_{f}$ are the mean and STD of the foreground/defect region, and $\overline{{\mu }_{b}}$ and ${\sigma }_{b}$ are the mean and STD of the background region. The selected areas as foreground and background regions are shown in Fig. 3(b1) by black and red rectangles, respectively.

## Supplementary information

Supplementary information.
