# Supplementary material for: Low-Cost Active Thermography using Cellphone Infrared Cameras: from Early Detection of Dental Caries to Quantification of THC in Oral Fluid
Source: Sci Rep. 2020 May 12;10:7857. doi: 10.1038/s41598-020-64796-6 (PMC7217835; doi:10.1038/s41598-020-64796-6)
Supplement: Supplementary file 1 — Supplementary information. [file 41598_2020_64796_MOESM1_ESM.docx]

**Low-Cost Active Thermography using Cellphone Infrared Cameras: from Early Detection of Dental Caries to Quantification of THC in Oral Fluid**

Nakisa Samadi^+^, Damber Thapa^+^, Mohammadhossein Salimi, Artur Parkhimchyk, and Nima Tabatabaei^*^

Department of Mechanical Engineering, Lassonde School of Engineering, York University, 4700 Keele St., Toronto, ON, M3J 1P3, Canada *Nima.Tabatabaei@Lassonde.YorkU.ca

^+^these authors contributed equally to this work


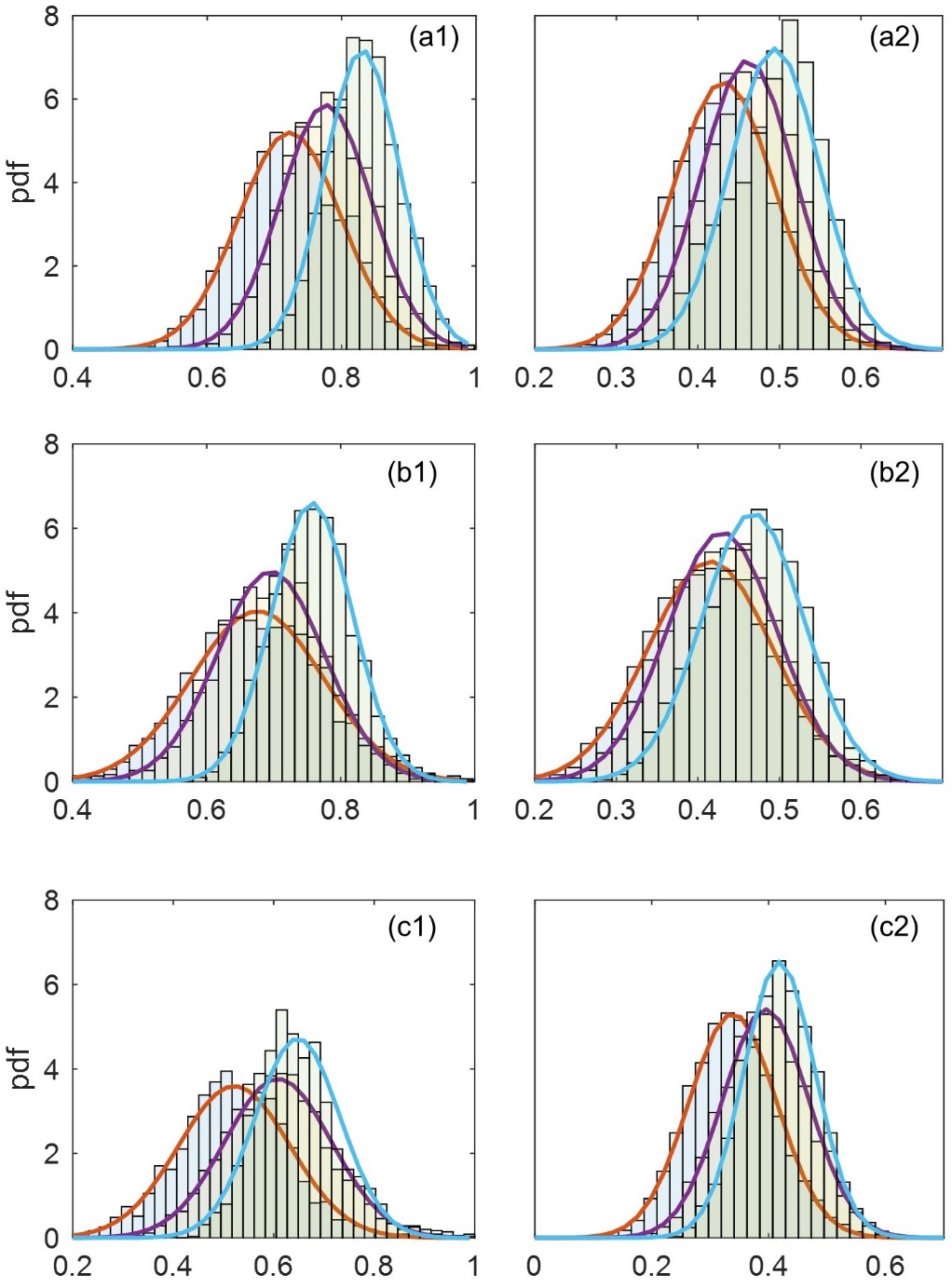


**Figure 1. Histograms with probability density function (pdf) of the images at the foreground (left) and background region (right) at camera frame rates of 9, 15 and 33 fps for defects at different depths. The panel a, b and c are corresponding to defects 300µm, 500µm and 800µm below intact interrogation surface, respectively. Mean ± STD of the pdf are reported in supplementary table 1.**

| Depth | Foreground | | | Background | | |
| --- | --- | --- | --- | --- | --- | --- |
|  | 9fps | 15 fps | 33 fps | 9fps | 15 fps | 33 fps |
| 300µm | 0.7230±0.0767 | 0.7767±0.0680 | 0.8308±0.0556 | 0.4318±0.0621 | 0.4612±0.0575 | 0.4941±0.0553 |
| 500µm | 0.6758±0.0988 | 0.6956±0.0802 | 0.7572±0.0604 | 0.4153±0.0765 | 0.4295±0.0675 | 0.4674±0.0627 |
| 800µm | 0.5215±0.1109 | 0.6086±0.1060 | 0.6476±0.0845 | 0.3388±0.0751 | 0.3950±0.0737 | 0.4186±0.0610 |

**Table 1. Means and STDs of the probability density functions calculated from the Supplementary Fig. 1.**
